# Characterization of Microbiota in Cancerous Lung and the Contralateral Non-Cancerous Lung Within Lung Cancer Patients

**DOI:** 10.3389/fonc.2020.01584

**Published:** 2020-08-24

**Authors:** Minglei Zhuo, Tongtong An, Chaoting Zhang, Ziping Wang

**Affiliations:** ^1^Key Laboratory of Carcinogenesis and Translational Research (Ministry of Education/Beijing), Department of Thoracic Oncology, Peking University Cancer Hospital & Institute, Beijing, China; ^2^Key Laboratory of Carcinogenesis and Translational Research (Ministry of Education/Beijing), Laboratory of Biochemistry and Molecular Biology, Peking University Cancer Hospital & Institute, Beijing, China

**Keywords:** microbiota, 16S rRNA, lung cancer, bronchoalveolar lavage fluid, cancerous lung and the contralateral non-cancerous lung

## Abstract

**Background:**

The functional role of lung microbiota has attracted an accumulating attention recently, but the profile and functional role of the lung microbiota in patients with lung cancer remained largely unknown.

**Methods:**

To evaluate the association of the microbiota with lung cancer, we performed comparative analysis of the lung microbiota using 16S rRNA amplicon sequencing approach in the paired bronchoalveolar lavage fluid (BALF) samples (paired samples from cancerous lung and the contralateral non-cancerous lung) from 50 cancer patients with unilateral lobar masses.

**Results:**

We found that the relative abundance of phylum Tenericutes, its class Mollicutes, its order Entomoplasmatales, its family Spiroplasmataceae, and its genus *Spiroplasma* was significantly increased in cancerous lung, but the relative abundance of phylum Bacteroidetes, its class Bacteroidia, and its order Bacteroidales was significantly decreased in cancerous lung. In addition, the relative abundance of family Leuconostocaceae and its genus *Weissella* was significantly increased in cancerous lung.

**Conclusion:**

Our findings provide insights into a change of lung microbiota community associated with the development of lung cancer.

## Introduction

Lung cancer is the leading cause of cancer-related morbidity and mortality worldwide (1). Multiple studies suggest that several factors, such as smoking (2), inflammatory response (3), and infections with several microbiota (4–6), are associated with lung cancer development.

Since mucosal tissue harbors the largest surface area in the human body, the lung is exposed to many airborne microbiota and environmental factors through inhalation. Thus, the relationship between the lung microbiota and lung disease is of particular interest. Healthy human lungs were traditionally deemed to be sterile owing to the inability of culturing bacteria from lower airway samples through routine microbiological methods (7). However, since the first culture-independent study found microbiota in asthmatic airways (8), series of studies using diverse molecular approaches have indicated that the healthy lung is also home to a complex diversity of microbiota (9, 10).

The functional role of respiratory microbiota has recently attracted an accumulating attention (11, 12) and additionally several studies found that variations of local microbial community were associated with the exacerbation of several respiratory disorders, including asthma, chronic obstructive pulmonary disease, and cystic fibrosis (7, 13–15).

However, the profile and functional role of the lung microbiota in patients with lung cancer remained largely unknown. To date, three studies used high-throughput sequencing for characterizing the lung microbiota in lung cancer patients, using bronchoalveolar lavage fluid (BALF) samples (16), protected specimen brush (17), and tissue samples (18), respectively. The two studies using BALF and protected specimen brush enrolled fewer patients (*n* < 30) (16, 17), and another study collected lung biopsy in non-cancerous tissue. Moreover, in healthy lungs, spatial variation of microbiota within an individual was significantly less than variation across individuals (19) and thus comparative analysis of the lung microbiota between lung cancer patients and healthy control subjects could produce inaccurate results regarding microbiota related with lung cancer.

To evaluate the association of the microbiota with lung cancer, we performed comparative analysis of the lung microbiota in the paired BALF samples (one from the cancerous lung lobe, the other from the contralateral corresponding non-cancerous lung lobe) from patients with lung cancer.

## Materials and Methods

### Subject Recruitment and Sample Collection

This study received ethical approval from the Institutional Review Board of the Peking University School of Oncology, China, and informed consent was obtained from all subjects. Fifty cancer patients with unilateral lobar masses were selected from patients consented to bronchoscopy examination at Beijing Cancer Hospital, Beijing, China. Demographic and clinical data, including age, gender, smoking, drinking, family history, and final diagnosis were obtained from each participant. Transbronchoendoscope, which avoided contamination of the upper respiratory tract or oral microbiota, was performed to obtain paired BALF samples in lung cancer patients (one from the cancerous lung, the other from the contralateral non-cancerous lung). All samples were immediately frozen and maintained at −80°C until further DNA extraction.

### DNA Extraction, 16S rRNA Amplification and Sequencing

Bacterial DNA was isolated from airway samples using PowerSoil DNA Isolation Kit (MoBio Laboratories, Carlsbad, CA, United States) following the manufacturer’s instructions. PCR was used with unique primers to amplify the V3–V4 region of the 16S rRNA to obtain an amplicon library from BALF samples, and amplicons were sequenced using the Miseq platform, as previously described (20).

### Sequence Data Analysis

Clean data were trimmed from raw data by trimmomatic 0.33. Operational taxonomic units (OTUs) were classified based on 97% similarity after chimeric sequences were removed using UPARSE (version 2.7.1). The phylogenetic affiliation of each 16S rRNA gene sequence was analyzed by RDP Classifier^1^ against the Silva (SSU128) 16S rRNA database using a confidence threshold of 70%. α-diversity (within-subject diversity) was assessed on the basis of the non-parametric Shannon Wiener diversity index and Simpson diversity index. The relationship between overall microbiota composition (β-diversity) and lung cancer was assessed by analysis of weighted and unweighted UniFrac distances calculated in QIIME (21). To visualize separation of samples based on pairwise distances, principal coordinate analysis (PCoA) plots were generated using the first three principal coordinates. The QIIME pipeline was also used to generate PCoA plots to visualize the unweighted UniFrac dissimilarity. We also broadly compared bacterial taxa (phylum to genus) between cases and controls. We limited our analysis of bacterial phyla to those with mean relative abundance ≥0.01%. For lower-level taxa (class to genus), we limited analysis to those with mean relative abundance ≥0.0001%. Paired *t*-test and Wilcoxon signed-ranks test were used with false discovery rate (FDR) to adjust *P*-values for multiple testing to detect taxa with differential abundance among groups. α-diversity difference between cancer and contralateral normal lung was examined in conditional logistic analysis. Bar plots and PCoA plots were all generated in R^2^.

### Accession Number

Raw sequencing data were submitted to the Sequence Read Archive (BioProject ID: PRJNA552813).

## Results

### Characteristics of the Study Participants

This study prospectively enrolled 50 patients, including 46 primary lung cancer patients and 4 patients with metastatic cancer in lung. The clinical characteristics of patients are listed in Table 1. In total, 34 males and 16 females were enrolled and additionally 29 smokers and 14 drinkers were included. The average age was 60 years old, and 9 patients have a family history of cancer. There were 14 cases (28%) diagnosed with lung squamous cell carcinoma, 12 cases (24%) with lung adenocarcinoma, 7 cases (14%) with non-specified non-small cell lung cancer, 13 cases (26%) with small cell lung cancer, 2 cases with colorectal cancer, 1 case with breast cancer, and 1 case with renal clear cell cancer. Among all patients, 5 patients (10%) were diagnosed in stages IA–IIIA and 44 patients (90%) had more advanced cancer stages (IIIB, IV) (Table 1). In 46 lung cancer patients, none previously had received chemotherapy, radiation therapy, or other treatments.

**TABLE 1:** Characteristics of subjects.

Variable	Value
**Age**	
Mean ± SD	60 ± 9.6
**Gender**	
Male	34
Female	16
**Smoking**	
Yes	29
No	21
**Drinking**	
Yes	14
No	36
**Histology type**	
Adenocarcinoma	12
Squamous carcinoma	14
Small cell lung cancer	13
NOS	7
Lung metastatic cancer	4
**AJCC 8th stage**	
IA	1
IIB	2
IIIA	2
IIIB	16
IV	28
**T stage**	
T1	2
T2	12
T3	18
T4	16
**Lymph node involvement**	
N0	2
N1	8
N2	15
N3	23
**Family history**	
Yes	9
No	41

*NOS: non-specified non-small cell lung cancer. AJCC: American Joint Committee on Cancer.*

### Taxonomic Profiles of the Cancer and Normal Lung Microbiota

**FIGURE 1:**
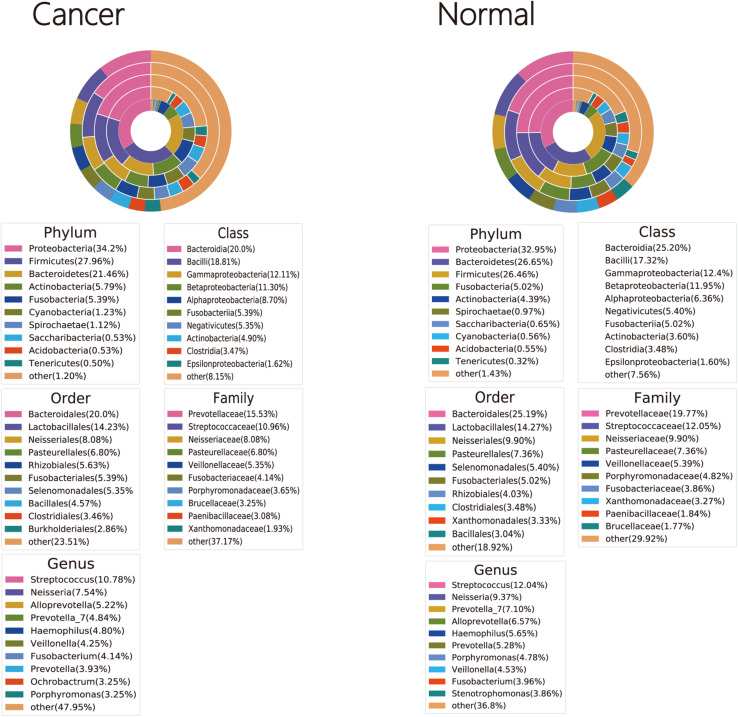
Taxonomic profiles of the cancer and normal lung microbiota.

Bacterial OTUs were classified into 46 and 43 phyla, 123 and 112 classes, 177 and 158 orders, 342 and 313 families, and 690 and 631 genera in cancer lung and normal lung samples, respectively (Supplementary Tables 2–6). Four phyla (Firmicutes, Proteobacteria, Fusobacteria, and Bacteroidetes) were detected in all cancer lung and normal lung samples. Seven genera (*Prevotella*_*7*, *Gemella*, *Porphyromonas*, *Haemophilus*, *Fusobacterium*, *Veillonella*, and *Streptococcus*) and 10 genera (*Neisseria*, *Porphyromonas*, *Fusobacterium*, *Campylobacter*, *Alloprevotella*, *Prevotella_6*, *Streptococcus*, *Veillonella*, *Haemophilus*, and *Prevotella_7*) were detected in all cancer lung and normal lung samples, respectively (Supplementary Tables 1–5). The three dominant phyla, classes, orders, and families were the same among cancer lung and normal lung samples. The two dominant genera were the same, but the third dominant genera were *Alloprevotella* and *Prevotella* among cancer lung and normal lung samples, respectively (Figure 1).

### Overall Microbiota Diversity in Relation to Cancer and Normal Lung

**FIGURE 2:**
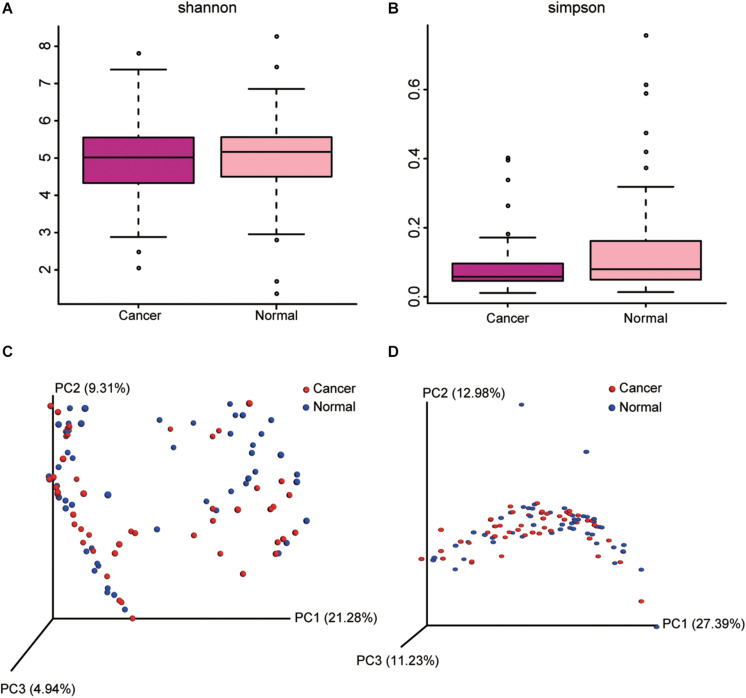
Comparison of alpha diversity metrics and beta diversity distance between cancer and normal lung microbiota. **(A,B)** Comparison of Shannon **(A)** and Simpson **(B)** index between cancer and normal lung microbiota is shown. **(C,D)** Ordination of the principal coordinates analysis performed on the unweighted **(C)** and weighted **(D)** UniFrac metric.

Cancer lung was not significantly different from normal lung in α-diversity (within-subject diversity), as measured by the Shannon (*P* = 0.871) and Simpson diversity index (*P* = 0.627), or overall lung microbiota composition (β-diversity, between-subject diversity), as measured by unweighted and weighted UniFrac distances (*P* = 0.952) (Figure 2).

### Comparison of Microbiota From Cancer and Normal Lung

**FIGURE 3:**
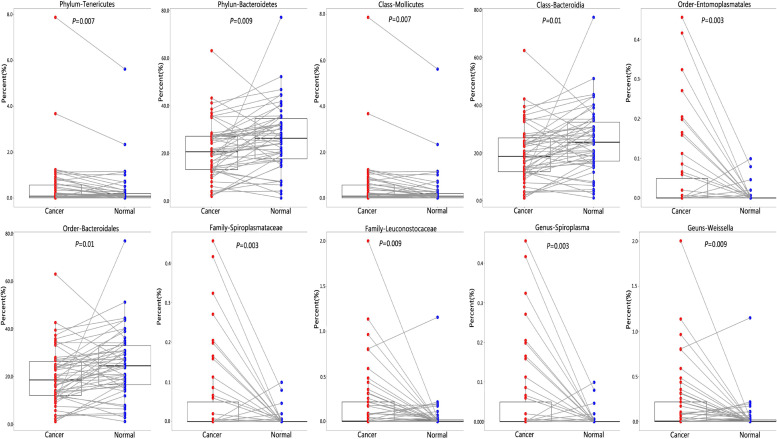
Difference of relative abundance of lung microbiota between cancer and normal lung. To identify differential microbial taxa, paired *t*-test was performed and *P-*values were adjusted for multiple comparison by the FDR.

We found that greater abundance of phylum Tenericutes (*P* = 0.007) was associated with increased risk of lung cancer, as well as its class Mollicutes (*P* = 0.007), its order Entomoplasmatales (*P* = 0.003), its family Spiroplasmataceae (*P* = 0.003), and its genus *Spiroplasma* (*P* = 0.003). The abundance of phylum Bacteroidetes was associated with reduced risk of lung cancer (*P* = 0.009), as well as its class Bacteroidia (*P* = 0.01), and its order Bacteroidales (*P* = 0.01). Family Leuconostocaceae (*P* = 0.009) and its genus *Weissella* (*P* = 0.009) were positively associated with lung cancer risk, although its phylum Firmicutes, class Bacilli, and order Lactobacillales were not significantly associated with lung cancer risk (Figure 3 and Supplementary Table 6).

## Discussion

Despite accumulating evidence linking microbiota to cancer, the role of lung microbiota in lung cancer has not been fully elucidated. In this study, we firstly characterized the taxonomic profiles of the microbiota in BALF from bilateral lungs (cancer lung and normal lung) within the same patients with lung cancer and found that increased relative abundance of phylum Tenericutes, its class Mollicutes, its order Entomoplasmatales, its family Spiroplasmataceae, and its genus *Spiroplasma* could be associated with lung cancer risk, but decreased relative abundance of phylum Bacteroidetes, its class Bacteroidia, and its order Bacteroidales could be related with risk of lung cancer. In addition, increased relative abundance of family Leuconostocaceae and its genus *Weissella* could be associated with lung cancer risk.

To date, three studies used high-throughput sequencing for characterizing the lung microbiota in lung cancer patients, using BALF samples (20 lung cancer patients) (16), protected specimen brush samples (24 lung cancer patients) (17), and tissue samples (31 lung cancer patients) (18), respectively. Since these studies enrolled less lung patients and used different sampling methods, their findings were quite different.

We found that three dominant phyla were Firmicutes, Proteobacteria, and Bacteroidetes in normal and cancer lung, similar to results of the above three studies (16). However, three dominant genera in normal and cancer lung in our study were similar to Lee et al.’s and Liu et al.’s results (16, 17) but different from Yu et al.’s findings (18), indicating that sampling methods could significantly affect microbial detection since our study’s sampling method was similar to Lee et al.’s and Liu et al.’s sampling methods but different from Yu et al.’s sampling method. Since lung biopsy in tumor tissue could be an invasive approach and moreover lung biopsy in non-cancerous tissue and healthy human subjects is not ethical, it is more convenient to detect lung microbiota using BALF or protected specimen brush.

We found that neither α-diversity nor β-diversity was significantly different between cancer and health lung, similar to Liu et al.’s findings (17). However, Lee et al.’s (16) and Yu et al.’s (18) studies reported significantly higher and lower α-diversity in cancer lung than normal lung, respectively. Similar to our study, Liu et al.’s study compared α-diversity and β-diversity between cancerous lesion and contralateral non-cancerous site within the same patient with lung cancer, but Lee et al.’s (16) study compared patients with lung cancer and patients with benign diseases and Yu et al.’s (18) study obtained lung tissue samples not BALF, which indicated that significant variation across different individuals and sampling methods could contribute to controversial findings regarding diversity of lung microbiota.

Among the above three studies (16–18), each study found specific microbiota associated with lung cancer, but none of these microbiota was repeatedly found to be associated with lung cancer in all these three studies, which could be attributed to small sample size and different sampling methods. We found that increased relative abundance of not only genus *Spiroplasma* but also its family, order, class, and phylum was significantly associated with the lung cancer risk, which could indicate their reliable associations, although these three previous studies did not find the significant association of these microbial taxa with lung cancer (16–18). Moreover, we found decreased relative abundance of phylum Bacteroidetes, its class Bacteroidia, and its order Bacteroidales in cancer lung, which was similar to the findings in Vogtmann’s study using oral wash (22). In addition, we found increased relative abundance of family Leuconostocaceae and its genus *Weissella* in cancer lung. Previous studies reported that infections with *Weissella* spp. primarily occurred in immunocompromised status and/or those with other underlying medical conditions (23), which could account for increased abundance of the genus *Weissella* in patients with lung cancer.

To date, our study had the largest sample of patients with lung cancer, included paired samples derived from cancer and normal lung within the same patients, and used a transbronchoendoscope, which prevented contamination of the upper respiratory tract or oral microbiota to obtain paired BALF. Although our study has the above advantages, it also has some limitations. First, it has a cross-sectional study design and therefore cannot identify a causal relationship between microbiota and lung cancer. Second, although our study had the largest sample of patients with lung cancer until now, the sample size was moderate and thus our study did not evaluate the influence of age, gender, smoking status, antibiotic use, and lung function on lung microbiota. Further large-scale studies are needed to investigate the role of microbiota in patients with lung cancer. Third, although we compared the lung microbiota in the paired BALF samples (paired samples from cancerous lung and the contralateral non-cancerous lung) to reduce variations of lung microbiota across individuals, the variation across individuals still makes it hard to infer whether differences within one individual really reflect causal relationship.

## Conclusion

In conclusion, our study found that there were differences in the microbial communities of cancer and normal lung within the same patient with lung cancer, and the genera *Spiroplasma* and *Weissella* were significantly enriched in cancer lung. These findings indicate that within individuals with unilateral lung cancers, there are differences in the microbiota between the cancerous and non-cancerous sides, but further large-scale studies and function of microbiota in animal model systems are essential to investigate potential use for prevention, diagnosis, and treatment strategies targeting the lung microbiota.

## Data Availability Statement

The datasets presented in this study can be found in online repositories. The names of the repository/repositories and accession number(s) can be found below: Sequence Read Archive (BioProject ID: PRJNA552813) (https://www.ncbi.nlm.nih.gov/bioproject/?term=PRJNA552813).

## Ethics Statement

The studies involving human participants were reviewed and approved by the Institutional Review Board of the Peking University School of Oncology. The patients/participants provided their written informed consent to participate in this study.

## Author Contributions

ZW and CZ designed the research. MZ, TA, and CZ conducted the experiments. CZ and MZ analyzed the data. CZ, ZW, and MZ wrote the manuscript. All authors contributed to the article and approved the submitted version.

## Conflict of Interest

The authors declare that the research was conducted in the absence of any commercial or financial relationships that could be construed as a potential conflict of interest.

## Footnotes

1 http://rdp.cme.msu.edu/

2 http://www.R-project.org/
